# Enhancing implementation of tobacco use prevention and cessation counselling guideline among dental providers: a cluster randomised controlled trial

**DOI:** 10.1186/1748-5908-6-13

**Published:** 2011-02-14

**Authors:** Masamitsu Amemori, Tellervo Korhonen, Taru Kinnunen, Susan Michie, Heikki Murtomaa

**Affiliations:** 1 Department of Oral Public Health, Institute of Dentistry, University of Helsinki, Helsinki, Finland; 2 Department of Public Health, Hjelt Institute, University of Helsinki, Helsinki, Finland; 3 Department of Oral Health Policy and Epidemiology, Harvard School of Dental Medicine, Harvard University, Boston, USA; 4 Centre for Outcomes Research and Effectiveness, Department of Clinical, Educational and Health Psychology, University College London, London, UK

## Abstract

**Background:**

Tobacco use adversely affects oral health. Tobacco use prevention and cessation (TUPAC) counselling guidelines recommend that healthcare providers ask about each patient's tobacco use, assess the patient's readiness and willingness to stop, document tobacco use habits, advise the patient to stop, assist and help in quitting, and arrange monitoring of progress at follow-up appointments. Adherence to such guidelines, especially among dental providers, is poor. To improve guideline implementation, it is essential to understand factors influencing it and find effective ways to influence those factors. The aim of the present study protocol is to introduce a theory-based approach to diagnose implementation difficulties of TUPAC counselling guidelines among dental providers.

**Methods:**

Theories of behaviour change have been used to identify key theoretical domains relevant to the behaviours of healthcare providers involved in implementing clinical guidelines. These theoretical domains will inform the development of a questionnaire aimed at assessing the implementation of the TUPAC counselling guidelines among Finnish municipal dental providers. Specific items will be drawn from the guidelines and the literature on TUPAC studies. After identifying potential implementation difficulties, we will design two interventions using theories of behaviour change to link them with relevant behaviour change techniques aiming to improve guideline adherence. For assessing the implementation of TUPAC guidelines, the electronic dental record audit and self-reported questionnaires will be used.

**Discussion:**

To improve guideline adherence, the theoretical-domains approach could provide a comprehensive basis for assessing implementation difficulties, as well as designing and evaluating interventions. After having identified implementation difficulties, we will design and test two interventions to enhance TUPAC guideline adherence. Using the cluster randomised controlled design, we aim to provide further evidence on intervention effects, as well as on the validity and feasibility of the theoretical-domain approach. The empirical data collected within this trial will be useful in testing whether this theoretical-domain approach can improve our understanding of the implementation of TUPAC guidelines among dental providers.

**Trial registration:**

Current Controlled Trials ISRCTN15427433

## Background

### Tobacco use prevention and cessation counselling among dental providers

Globally, tobacco use remains the leading preventable risk factor for premature morbidity and mortality [1]. Tobacco use is harmful to all human biological systems, including the oral cavity. It is a major contributor to oral cancer and periodontal diseases and is a significant risk factor for failed dental implant therapy [2-4]. Other effects relevant to dentistry are staining and discolouration of teeth and dental restorations, as well as congenital defects such as oral clefts if expectant mothers smoke [4-6]. Conversely, tobacco use cessation has positive immediate and long-term effects; smell and taste return to normal within one month after cessation, while the risk for oral cancer, for example, decreases to nearly the same level as for never-users during the following years [2,4].

In Finland, primary healthcare is provided by municipal health centres under the Primary Health Act. This also includes free or financially subsidised dental care. Health promotion and prevention are the main responsibilities of health centres and are becoming increasingly important as healthcare costs are growing. Currently, the Finnish government and municipal administrations are working to develop health centres' operations towards more cost-effective practices (The Government Resolution on the Health 2015 public health programme, http://pre20031103.stm.fi/english/eho/publicat/health2015/health2015.pdf). To improve the quality of care, as well as the cost-effectiveness of primary care, healthcare professionals should be better supported in implementing clinical guidelines and preventive services.

Annually, more than one-third of Finnish residents visit a dental practitioner in health centres, with an average of 2.6 appointments per year [7]. This gives an excellent opportunity for dental providers to make a high public health impact, for example, in tobacco cessation. The fact that over 80% of tobacco users are worried about the health effects of smoking and some 60% would like to give it up [8] shows the potential for dental providers to contribute to tobacco use prevention and cessation (TUPAC) counselling. Besides cessation, promoting tobacco abstinence is particularly important among young people who are likely to take up tobacco use. In Finland, dental providers in health centres meet about 75% of the population of minors (<18 years) each year [7], more than other healthcare professionals. This opportunity has been recognised by the World Health Organization (WHO) Global Oral Health Programme, the European Union (EU) Working Group on Tobacco and Oral Health, and recently by the European Workshop on Tobacco Use Prevention and Cessation for Oral Health Professionals [9-11]. The primary message is that oral health professionals should strengthen their contributions to tobacco cessation programmes so that all patients who use tobacco are counselled to quit.

### Guidelines on tobacco dependency treatments

The Finnish Medical Society Duodecim produces national Current Care guidelines based on up-to-date evidence to support healthcare decision making in Finland. The guideline for Smoking, Nicotine Addiction, and Interventions for Cessation was published for the first time in 2002 and updated in 2006. The Current Care guidelines for TUPAC counselling recommend a six *A*s approach (Ask, Assess, Account, Advise, Assist, Arrange) [12], which is similar to the five *A*s approach presented by US and other national guidelines [13]. The main principles in TUPAC guidelines include a recommendation that the healthcare provider ask about each patient's tobacco use at least once a year, assess the patient's readiness and willingness to stop, document tobacco use habits (what type of tobacco, quantity, duration), advise the patient to stop tobacco use and instigate supportive measures where necessary, assist and help the patient in his/her attempt to stop tobacco use, and arrange monitoring of progress at follow-up appointments. Historically, however, dental providers, and dentists in particular, have not been routinely involved in the TUPAC counselling. The latest national data show that only 10.5% of daily tobacco users who had visited a dentist during the past year had received advice to quit tobacco use [8]. The gap between guideline recommendation and implementation is evident.

### Developing interventions to enhance guideline implementation

The challenges in designing interventions to increase healthcare providers' effective implementation of clinical guidelines are many. Although the implementation depends on behaviour change, much of the current research investigating methods of increasing guideline implementation does not draw on theories of behaviour change. The UK's Medical Research Council (MRC) has produced guidance for designing and evaluating interventions that emphasises the importance of applying theory to the early phases of intervention development [14]. Examples of such theories are the Theory of Planned Behaviour [15], Social Cognitive Theory [16], and Theory of Interpersonal Behaviour [17]. Since many theories exist, it is often unclear which theory to use in addressing an implementation problem. To simplify the selection of theory, a consensus group of health psychologists and implementation researchers identified 12 theoretical domains from 33 theories of behaviour change that could be used to investigate the implementation of clinical guidelines [18]. These are knowledge; skills; professional role and identity; beliefs about capabilities; beliefs about consequences; motivation and goals; memory, attention, and decision processes; environmental context and resources; social influences; emotion; behaviour regulation; and nature of behaviours. This theoretical-domains framework provides a comprehensive basis for assessing problems and will serve as the first key step in our study to evaluate implementation difficulties of TUPAC guidelines among dental providers.

**Figure 1:**
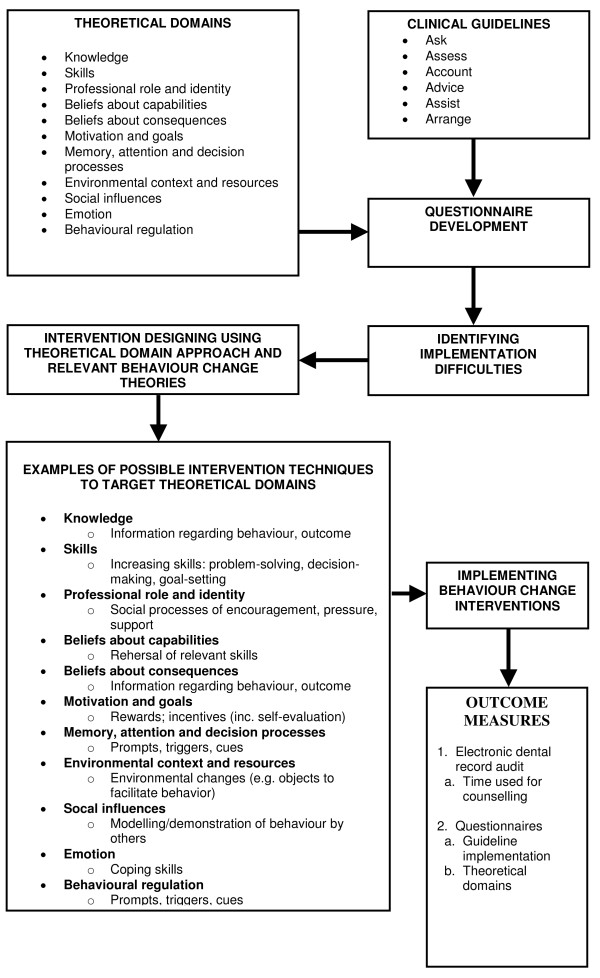
**Steps for modelling intervention (modified from Medical Research Council framework) **[14].

To progress from a theoretical assessment of the implementation problem to intervention design, Michie *et al. *have proposed a list of behaviour-change techniques to target each of the theoretical domains [19], examples of which are shown in Figure 1. For example, if the domain motivation and goals needs improvement, behaviour-change techniques such as rewards, graded tasks, and motivational interviewing would be suitable intervention components. If beliefs about consequences need changing, providing information regarding intervention outcomes could be used. Thus, the theoretical framework can guide the selection of behaviour-change techniques in enhancing guideline adherence among healthcare providers.

### Aims and objectives

The general aim is to enhance implementation of TUPAC counselling guidelines among dental providers. The first objective is to develop a theoretically informed measure for assessing the implementation difficulties among dental providers related to TUPAC counselling guidelines (six *A*s approach) using a theory-based assessment and to apply it to a sample of Finnish dental providers. After identifying implementation difficulties, our second objective is to design two interventions to enhance guideline adherence using relevant behaviour-change theories and intervention techniques. Finally, we aim to conduct a cluster randomised controlled trial to assess intervention effects. A cluster design will be used to reduce contamination across participants.

The theoretical and chronological framework of the study is provided in Figure 1.

## Methods

### Participants

**Figure 2:**
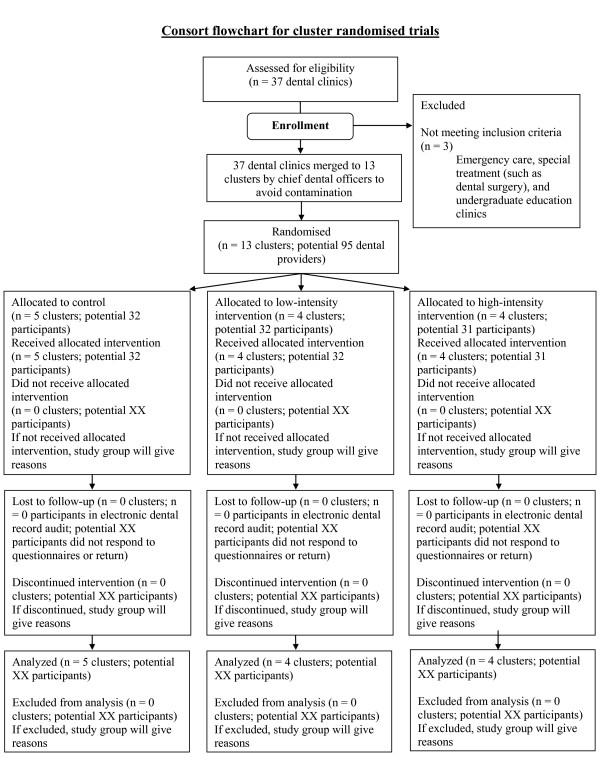
**Potential flowchart of participants and clusters in OH NO TOBACCO! trial**.

All dentists and dental hygienists employed by the Finnish municipal health centres of Vaasa (9 clinics) and Tampere (28 clinics) will be invited to participate, except two clinics' staff in Tampere (emergency and special treatment clinic) and one clinic's staff in Vaasa (undergraduate education clinic) (Figure 2). Implementing TUPAC counselling interventions in those excluded clinics would not be feasible. Participants meeting the inclusion criteria will receive the explanatory statement of the study (additional files 1 and 2), consent form (additional file 3), and instructions to participate (additional file 4). The survey will be conducted using either a web-based survey http://www.surveymonkey.com or a more traditional paper form survey. Strategies to promote response rates among dental providers include offering two movie tickets (value about €10 per ticket) for participation. We will also send two reminder letters (the first reminder one week and the second two weeks after the first request to respond) to nonrespondents.

### Primary outcome measures

The meta-analysis shows that if TUPAC guidelines are implemented, the time used by healthcare providers for counselling is one of the best predictors for counselling success [13]. As our target behaviour will be the implementation of TUPAC guidelines, our primary outcome measures will be (a) whether the TUPAC guideline recommendations are implemented, and (b) if implemented, the estimated time used for the counselling. We will use the electronic dental record (EDR) audit for measuring these outcomes. If the dentist or dental hygienist provides TUPAC counselling, documented procedure codes will give information on the effect counselling may have had. A similar procedure-code documenting system is widely used in dentistry (fillings, extractions, etc.). The codes for TUPAC counselling will be as follows: TI02 = minimal counselling (<3 minutes), TI03 = low-intensity counselling (3 to 10 minutes), and TI04 = higher-intensity counselling (>10 minutes). Categories of intervention duration are based on the meta-analysis, where the estimated odds ratios (ORs) for TUPAC counselling are reported using the same counselling durations (OR = 1.3 for minimal counselling, OR = 1.6 for low-intensity counselling, and OR = 2.3 for higher-intensity counselling) [13]. When multiplying the procedure codes by the estimated ORs and summing the results, we will create one continuous primary outcome. The EDR softwares used in the Vaasa and Tampere health centres are identical (Effica by Tieto Finland, Helsinki) and include the above-mentioned codes for each intervention intensity.

### Secondary outcome measures

In order to identify implementation difficulties of TUPAC counselling guidelines among dental providers, a Theoretical Domain Questionnaire (TDQ) will be developed according to the theoretical framework published by Michie *et al. *[19]. Additionally, the TDQ will be based on the Finnish Current Care guidelines on TUPAC counselling (six *A*s approach). We will select items from published literature and create new items to cover different aspects of the guideline recommendation and theoretical domains. The aim of the TDQ development is to create a tool to assess the mediators and theoretical explanations for implementation difficulties.

Adherence to the TUPAC counselling guidelines will be assessed by a previously used and validated instrument [20,21] covering the six *A*s approach [12]. A similar questionnaire will be developed for patients to receive more objective results of dental providers' implementation of TUPAC guidelines. For determining participants' tobacco use, derivation of smoking index will be used (additional file 5).

### Trial design

After developing the TDQ, we will conduct a provider baseline survey and EDR audit to measure the baseline adherence to TUPAC counselling guidelines and prevailing implementation difficulties among our sample. Based on identified implementation difficulties, we will use relevant behaviour-change theories and techniques in designing two interventions to enhance TUPAC guideline implementation. In selecting relevant intervention techniques, we will use a matrix of theoretical domains and 35 behaviour-change techniques [19] (Figure 1). Finally, we will test these interventions using a cluster randomised controlled trial (Figure 2).

Dental providers usually work in only one clinic, but when this is not the case, chief dental officers will merge two or more clinics into one cluster to reduce contamination across participants. After merging clinics and forming clusters, chief dentists will provide a concealed sequence of clusters to investigators who will allocate clusters randomly to (a) control, (b) low-intensity intervention, or (c) high-intensity intervention groups (Figure 2) by drawing lots. Allocation will be concealed from the investigators until data collection has been conducted. Investigators, patients, outcome assessors, and study statistician will be blinded to group allocation until the statistical analysis has been completed. Due to the nature of the study setting, it is not possible to blind the dental providers for group allocation. The success of blinding will not be evaluated.

### Sample size

There is a scarcity of recent national data regarding the implementation of TUPAC counselling guidelines reported by dental providers. Hence, we conducted our sample size calculations based on population reports collected by the National Institute for Health and Welfare from a random sample (n = 5,000) of Finnish adults [8]. The data showed that 10.5% of surveyed tobacco users who visited the dentist at least once during the past 12 months had received any TUPAC counselling [8]. As our primary aim is to compare the implementation of TUPAC counselling guidelines between control versus two intervention groups, sample size is calculated using the following assumptions: Our aim is to increase the proportion of counselled patients from 10.5% (control) to 33% in the first (low-intensity) intervention group and to 63% in the second (high-intensity) intervention group, as validated by dental record audit. To achieve 80% power, with a two-sided 5% significance level with an estimated intra-class correlation of 0.02, we will need totally 72 participants and 12 clusters with an average of six participants per cluster. Assuming a baseline response rate of 76%, we will need a sample of 95 dental providers.

### Data analysis

We will follow intention-to-treat principles at both individual and cluster levels. Participants will be assigned to the cluster they were in when the trial began. However, if a participant moves to another cluster during the trial period that is assigned to a higher intervention arm, they will be shifted to that cluster.

In data analysis, we will first analyse descriptive variables to explore the distribution of background data using chi-square and *t*-tests. To compare intervention effects between control and intervention groups, we will use adjusted, generalised linear models and modified *t*-tests, taking into account the effect size. Analyses will be conducted at the cluster and individual level, and all estimates will be presented with standard deviations or 95% confidence intervals.

### Ethical review

The Ethics Committees of the Pirkanmaa Hospital District and Vaasa Central Hospital have approved our research plan. The permission to conduct the study was received from the Research Permission Committee of the City of Tampere and the medical director of the Vaasa health centre.

### Trial update

The baseline survey (background information, self-reported guideline implementation, the theory-based assessment of implementation difficulties) and the EDR audit of the sample were conducted in September 2009. Of those eligible, 76.8% participated (n = 73/95). The study participants were fairly representative of municipal dental providers (Table 1). Participating dentists had practiced more clinical years on average (22.4 years) compared to dental hygienists (10.2 years; *p *< .001) and reported higher lifetime tobacco abstinence (72.2%) than dental hygienists (21.1%; *p *< .001) (Table 2). Regular tobacco use was uncommon in both provider groups. More dental hygienists had received undergraduate education on TUPAC counselling compared to dentists (84.2% versus 24.1%; *p *< .001). The results of the self-reported guideline implementation, theory-based assessment of implementation difficulties, and EDR audit will be reported elsewhere.

**Table 1:** The comparison of gender and mean age of study participants, nonrespondents, and municipal dental practitioners in Finland

	Participants		Nonrespondents		Total		Municipal dentists
	Dentists (n = 54)	Hygienists (n = 19)	Dentists (n = 19)	Hygienists (n = 3)	Dentists (n = 73)	Hygienists (n = 22)	Dentists* (n = 2,002)
Female (%)	81.5	100	68.4	100	78.1	100	77.4
Mean age, years (SD)	48.7 (9.1)	37.3 (9.5)	51.1 (9.3)	46.7 (16.7)	48.9 (9.5)	38.6 (10.7)	49.5 (8.7)

*Finnish Dental Association statistics 2010. No national data available for dental hygienists.

**Table 2:** Participant characteristics at baseline

	Dentists (n = 54)	Dental hygienists (n = 19)	*p *value*	Total (n = 73)
Response rate (%)	74.0	86.4	.27	76.8
Years practised (SD)	22.4 (9.1)	10.2 (7.6)	<.001	19.2 (10.2)
Mean clinical hours per week (SD)	28.0 (7.4)	31.1 (8.2.)	.14	28.8 (7.7)
Tobacco use (%)				
Never	72.2	21.1	<.001	58.9
Gave up 1 to 12 months ago	3.7	0	.40	2.7
Occasional	1.9	10.5	.10	4.1
Regular	3.7	5.3	.71	4.1
Received undergraduate education on tobacco use prevention or cessation counselling (%)	24.1	84.2	<.001	39.7
Received continuing education on tobacco use prevention or cessation counselling (%)	37.0	31.6	.67	35.6

*p values calculated using chi-square and *t*-tests.

## Discussion

The present study protocol adopts a theory-based, step-by-step approach to investigating and enhancing implementation of TUPAC guidelines among dental providers. To our best knowledge, this is one of the first times that the theoretical-domain approach [18] will be used systematically both in development and evaluation of implementation research. As noticed by Berwick [22], it is important to understand not only whether interventions work but how and under what circumstances. Thus, using the theoretical-domain approach and EDR audit, we aim to evaluate the effectiveness of implemented interventions compared to the control group and provide explanations for how and why implemented interventions were effective or not. In addition, our trial may lead to recommendations for potentially effective strategies to enhance implementation of TUPAC guidelines.

Some limitations need to be addressed. As we will conduct the trial in community dental settings, contamination effects of interventions are possible. Although we will not inform participants about other intervention conditions, it is likely that dentists and dental hygienists will discuss the interventions with their colleagues during the study period. In order to minimise contamination, we need to conduct randomisation at the cluster level (*i.e.*, at the dental clinic level). We believe that the advantages associated with randomising dental clinics rather than dental providers will outweigh its disadvantages, such as loss of power. Second, as we are planning to collect our primary outcomes using electronic records (EDR), this may lead to underestimation of provided TUPAC counselling because dental providers may not always enter procedure codes, even if they have provided TUPAC counselling. Videotaping the consultations, for example, would enable us to more precisely evaluate the content and quality of the TUPAC counselling but would not be feasible, as it would influence provider behaviour. Third, theoretical-domain approaches do not, *per se*, identify the causal processes leading to behaviour change. However, our study is not an attempt to replace theories but to identify barriers, provide relevant explanations for implementation difficulties, and provide an evidence base for designing interventions. Although potentially useful, the TDQ will not demonstrate all factors that contribute to implementation of TUPAC counselling guidelines among dental providers, since length constraints preclude measuring all aspects of each domain and selecting the key point of each. Finally, even if our baseline response rate is high (76.8%) and our sample of dentists well represents the population (Table 1), our sample size is relatively small; a larger sample would provide greater power and better accuracy.

Despite possible limitations, the results of this trial will be relevant for decision makers and managers facing the challenge of implementing TUPAC guidelines among healthcare providers. In addition, this research constitutes a major contribution in using a theoretical-domain approach in implementation research. Although based on Finnish community dental settings and TUPAC guidelines, this theory-based approach may provide an important evidence base for future implementation research in different settings and professional disciplines.

## Competing interests

The authors declare that they have no competing interests.

## Authors' contributions

MA, TK, THK, and HM conceived the study and acquired funding. MA (principal investigator) conducted the data analysis, wrote the first draft of the manuscript, and reviewed and approved the final draft. SM was theoretical and methodological advisor. All authors advised on clinical and methodological issues, provided ongoing critique, and have approved the final version of the manuscript.

## Supplementary Material

Additional file 1 **Explanatory statement of the study, Tampere**. Click here for file

Additional file 2 **Explanatory statement of the study, Vaasa**. Click here for file

Additional file 3 **Study consent**. Click here for file

Additional file 4 **Instruction form for completing the survey**. Click here for file

Additional file 5 **The derivation of smoking index according to national health behaviour and health survey **[8]. Click here for file
