# Supplementary material for: Enhancing implementation of tobacco use prevention and cessation counselling guideline among dental providers: a cluster randomised controlled trial
Source: Implement Sci. 2011 Feb 14;6:13. doi: 10.1186/1748-5908-6-13 (PMC3055178; doi:10.1186/1748-5908-6-13)
Supplement: Additional file 1 — Explanatory statement of the study, Tampere. [file 1748-5908-6-13-S1.PDF]

HELSINGIN YLIOPISTO
HELSINGFORS UNIVERSITET
UNIVERSITY OF HELSINKI

OH-NO-TOBACCO!

DEAR RECIPIENT

We are conducting a survey on the promotion of tobacco abstinence and cessation by dental staff in the health centre of Tampere and kindly request your participation. The Ethics Committee of the Pirkanmaa Hospital District and the Research Permission Committee of the City of Tampere have approved our research plan.

Our survey examines the support and counselling that you provide to your patients to promote tobacco abstinence and cessation. To obtain reliable results, it is important that as many of the selected respondents as possible complete this questionnaire. The data will be used statistically, and no individual responses can be identified. Participation is voluntary and you may refuse to participate without giving a reason.

The survey consists of three questionnaires. Please respond to the first questionnaire according to the enclosed instructions. You will receive written instructions for follow-up questionnaires at a later date.

Please return the consent form in the enclosed postage-paid envelope. For your participation, you will receive **two Finnkino film tickets (worth 20 €) per questionnaire**. The tickets will be mailed to you within one month of receiving your completed questionnaire.

Every response is important to the further development of Finnish dental healthcare. Should you have any questions or require additional information, please do not hesitate to contact us.

Thank you for your participation.

Professor Heikki Murtomaa
Institute of Dentistry
University of Helsinki
heikki.murtomaa@helsinki.fi
(09) 19127264

Masamitsu Amemori, BDent
Institute of Dentistry
University of Helsinki
masamitsu.amemori@helsinki.fi
(09) 19129203

Contact person in Tampere:

Eeva Torppa-Saarinen
Chief Dental Officer
eeva.torppa-saarinen@tampere.fi
(03) 565 713 (switchboard)
