# Supplementary material for: Enhancing implementation of tobacco use prevention and cessation counselling guideline among dental providers: a cluster randomised controlled trial
Source: Implement Sci. 2011 Feb 14;6:13. doi: 10.1186/1748-5908-6-13 (PMC3055178; doi:10.1186/1748-5908-6-13)
Supplement: Additional file 3 — Study consent. [file 1748-5908-6-13-S3.PDF]

HELSINGIN YLIOPISTO
HELSINGFORS UNIVERSITET
UNIVERSITY OF HELSINKI

OH-NO-TOBACCO!

SURVEY OF TOBACCO USE AND ORAL HEALTH

I have received written information about the survey on the promotion of tobacco abstinence and cessation by dental staff, and have been provided with the opportunity to ask the researchers questions.

I understand that participation in the survey is voluntary and that I can refuse to participate at any time without giving a reason. I also understand that all information provided here will be kept confidential.

**I consent to participate in the survey.**	**Recipient of consent**
	Helsinki, 21 August 2009
**Place, date and signature**	**Place, date and recipient's signature**
	Professor Heikki Murtomaa
**Name in block letters**	**Recipient's name in block letters**

Please provide your contact details for a follow-up questionnaire.

Street address: ____

Postal code: ____________________

City or town: ____________________

Email address: ________________________________

I will respond online. ☐
I wish to receive a printed questionnaire. ☐
