# Supplementary material for: Enhancing implementation of tobacco use prevention and cessation counselling guideline among dental providers: a cluster randomised controlled trial
Source: Implement Sci. 2011 Feb 14;6:13. doi: 10.1186/1748-5908-6-13 (PMC3055178; doi:10.1186/1748-5908-6-13)
Supplement: Additional file 4 — Instruction form for completing the survey. [file 1748-5908-6-13-S4.PDF]

HELSINGIN YLIOPISTO
HELSINGFORS UNIVERSITET
UNIVERSITY OF HELSINKI

OH-NO-TOBACCO!

# INSTRUCTIONS FOR COMPLETION OF THE QUESTIONNAIRE

**1. Please proceed to the online questionnaire:**

www.helsinki.fi/hammas/tupakka

**2. Please select the following questionnaire:**

Staff questionnaire: Baseline

**3. Once you have selected the questionnaire, you will be asked for a password. Please enter the following password:** sondi

**4. The first question applies to your personal code.**

Please enter **carefully** the five-digit code at the bottom of the enclosed bar code label into the response field.

**Personal code: XXXXX**

**5. Please respond carefully to all questions.**

**6. When you have finished, please click on Submit.**

The questionnaire will take approximately 5–10 minutes to complete.

Should you prefer a printed questionnaire, please select “I wish to receive a printed questionnaire” on the consent form and enter your contact details. We will then mail the printed questionnaire to your mailing address; you need not complete the online questionnaire.
