# Supplementary material for: Enhancing implementation of tobacco use prevention and cessation counselling guideline among dental providers: a cluster randomised controlled trial
Source: Implement Sci. 2011 Feb 14;6:13. doi: 10.1186/1748-5908-6-13 (PMC3055178; doi:10.1186/1748-5908-6-13)
Supplement: Additional file 5 — The derivation of smoking index according to national health behaviour and health survey [8]. [file 1748-5908-6-13-S5.PDF]

Tupakointi-indeksin muodostaminen / Derivation of smoking index

Luokkien merkitys/Key to index classes:

1. Päivittäin tupakoivat/Daily smokers
2. Satunnaisesti tupakoivat/Occasional smokers
3. 1 – 12 kk sitten päivittäisen tupakoinnin lopettaneet/
   Quitters given up smoking 1-12 months ago
4. Yli vuosi sitten päivittäisen tupakoinnin lopettaneet/
   Ex-smokers given up daily smoking more than one year ago
5. Tupakoimattomat/Non-smokers
6. Riittämättömät tiedot/Insufficient information
   * Tieto puuttuu/Information missing

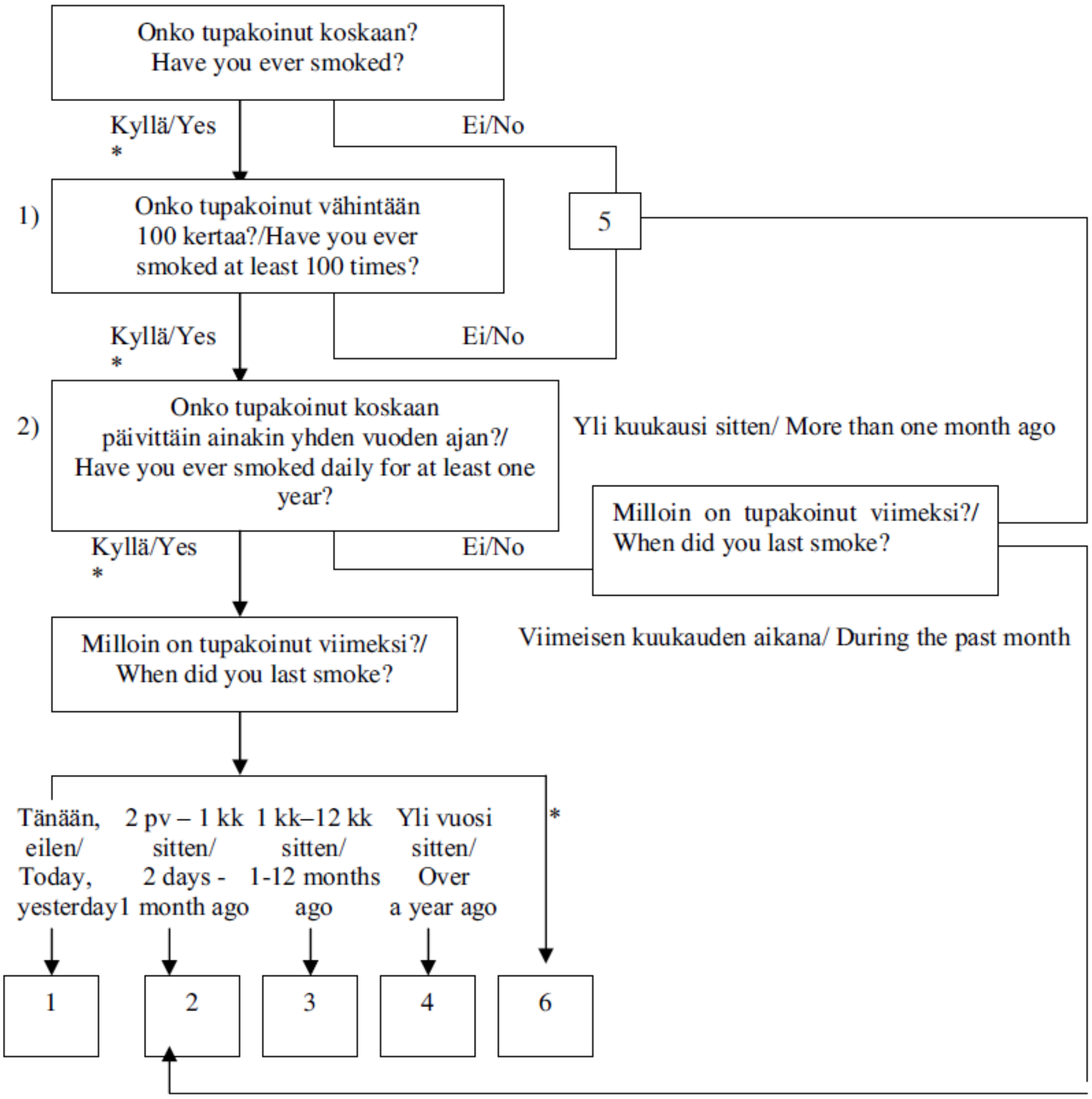

1) Kysymys lisätty vuonna 1996/Question introduced in 1996

2) Vuosina 1978 – 95 kysytty säännöllistä tupakointia. Vuodesta 1996 lähtien kysytty päivittäistä tupakointia/ During 1978 - 95 only regular smoking has been inquired. Since 1996 only daily smoking has been inquired.
